# Muscle Physiopathology in Parathyroid Hormone Disorders

**DOI:** 10.3389/fmed.2021.764346

**Published:** 2021-10-22

**Authors:** Cecilia Romagnoli, Maria Luisa Brandi

**Affiliations:** ^1^Department of Experimental and Clinical Biomedical Sciences “Mario Serio”, University of Florence, Florence, Italy; ^2^F.I.R.M.O. Italian Foundation for the Research on Bone Diseases, Florence, Italy

**Keywords:** skeletal muscle, PTH, parathyroid hormone disorders, satellite cells, skeletal muscle regeneration, parathormone

## Abstract

Parathyroid hormone disorders are a group of diseases in which secretion of parathormone (PTH) is impaired. The disorders that result are characterized by signs and symptoms associated with the persistent presence of high blood calcium levels (hypercalcemia) related to hyperparathyroidism (PHPT), or reduced blood calcium levels (hypocalcemia) associated with hypoparathyroidism (HypoPT). In addition to the resulting alteration in bone microarchitecture and mass for both pathologies, patients also report problems with skeletal muscle due to a decrease in muscular strength, muscular dysfunction, and myopathies, which can be responsible for an increased risk of instability and fracture. Although the effect of PTH on bone is well established, and numerous studies suggest that PTH has an effect on skeletal muscle, knowledge about cellular e molecular mechanisms of action on skeletal muscle is very limited. Skeletal muscle is a tissue well known for its structural and mechanical actions and is endowed with an extraordinary ability to adapt to physiological changes. Research in skeletal muscle has increased over the last decade, its importance as an endocrine tissue also emerging, becoming itself a target of numerous substances and hormones. Parathyroid hormone disorders represent a starting point to understand whether PTH may have an effect on skeletal muscle. This review analyzes the basic research data reported to date on PTH and skeletal muscle, highlighting the importance of increasing our knowledge in this field of research.

## Introduction: The Multifaceted Skeletal Muscle

Skeletal muscle is one of the most dynamic tissues of the human body, fundamental for vital functions like locomotion, postural support, breathing, and thermogenesis (1). It comprises ~35–45% of total body mass and, in general, muscle mass depends on the balance between protein synthesis and degradation, the processes of which are influenced by factors such as hormonal balance, physical activity and exercise, nutritional status, injury, and disease (2).

Skeletal muscle is an organ endowed with a remarkable ability and plasticity to adapt to physiological changes, such as growth, exercise, and tissue damage. This amazing capacity is attributable to a small population of mononuclear cells, which represent from 2 to 10% of all nuclei of a given fiber in healthy adult mammalian muscle, identified in 1961 by Alexander Mauro, named satellite cells (SCs), located between the plasma membrane of the myofibers and the basement membrane (sarcolemma) (3). SCs can be recognized by the presence of paired box protein 7 (PAX-7) a nuclear transcription factor which specifies the myogenic properties of muscle stem cells and act as a nodal factor by stimulating proliferation and inhibiting differentiation (1). In healthy unstressed conditions, SCs are mitotically quiescent (G_0_ phase) and transcriptionally inactive; after injury or degeneration, quiescent SCs rapidly re-enter the cell cycle, become activated, and proliferate to supply myoblasts. After activation and the proliferation phase, the expression of PAX-7 decreases, myoblasts exit the cell cycle and differentiate into mature muscle cells, fusing into existing myofibers to repair, or fuse together, to generate large numbers of new multinucleated myofibers within just few days (4).

**Figure 1:**
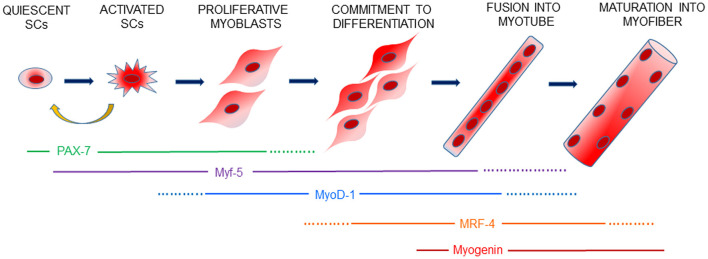
Schematic view of myogenesis. Skeletal muscle regeneration is finely controlled by a genetic cascade involving PAX-7 and the myogenic regulatory factors, Myf-5, MyoD-1, MRF-4 and Myogenin, which drive every step of SCs activation, proliferation of myoblasts and the differentiation and formation of new muscle fibers. Activated SCs can retain PAX-7 expression and return to a quiescent state to contribute to the replenishment of the SCs pool for future muscle regeneration.

Skeletal muscle regeneration (Figure 1) is a highly orchestrated process in which specific transcription factors, including Myf-5, MyoD-1, Myogenin and MRF-4, belonging to the basic helix-loop-helix (bHLH) family, are an essential group of muscle-specific proteins, called Myogenic Regulatory Factors (MRFs), responsible for acting at multiple time points in the muscle lineage to cooperatively establish the skeletal muscle phenotype (5). Moreover, like stem cells, SCs self-renew to maintain their own population, re-establishing their numbers and quiescent state by homing back to highly specialized niches, thus allowing future regeneration (6).

Research focused on skeletal muscle has greatly increased over the last decade. Skeletal muscle is also considered a secretory organ, capable of producing several substances of protein nature, called myokines, which can act in an autocrine, paracrine or endocrine fashion, on the muscle itself, on nearby tissues, or distant organs (7). These functions can be physiologically modulated by physical stimuli and by several hormones, or cytokines and mineral ions, or can be modified by endocrinopathies, sarcopenia, and/or morphological modification of muscle fibers (8). Moreover, the production of myokines makes them an excellent indicator of alterations in the endocrine activity of the skeletal muscle. In fact, since myokines are circulating factors that influence the direct muscle environment, but also other organ systems, they can contribute to understanding the physiological crosstalk between muscles and other organs, as well as the development of diseases and their prevention (9–11).

Considering the importance of healthy skeletal muscle to provide stability and strength for all the body, any alteration in its function leads to some degree of instability and/or impairment. Therefore, factors that influence skeletal muscle structure and function are a subject of great scientific and clinical interest.

Parathyroid hormone diseases, characterized by alteration of parathyroid hormone levels in the blood, are disorders that feature bone abnormalities and decreased skeletal muscle strength. This review will consider these disorders and take into consideration the current knowledge on the effects of PTH on skeletal muscle.

## Parathyroid Hormone Disorders and Associated Symptoms

Disorders of the parathyroid glands, 4 endocrine glands located on the dorsal part of the thyroid, most typically present with abnormalities of serum calcium concentration. The disorders that result are characterized by signs and symptoms associated with the persistent presence of high blood calcium levels (hypercalcemia) related to primary hyperparathyroidism (PHPT), or reduced blood calcium levels (hypocalcemia) associated with hypoparathyroidism (HypoPT) (12).

PHPT is an endocrine disorder, characterized by hypercalcemia combined with inappropriately normal or elevated parathyroid hormone (PTH) levels, which are associated with serious skeletal and renal complications. It is caused by excessive synthesis and secretion of PTH by one or more of the four parathyroid glands and leads to a reduction of bone mineral density (BMD), deterioration of bone microarchitecture, and increased risk of fractures, given the catabolic action of high levels of PTH on bone. Patients also present complex symptoms of muscular fatigue, myopathy, various neuropsychiatric and cardiovascular manifestations, and kidney stones (13, 14). In particular, muscle strength and quality of life (QoL), also impaired in PHPT, may contribute to increased fracture risk, independent of BMD (15). The classical treatment for PHPT is parathyroidectomy with the surgical removal of hyper-functioning parathyroid tissue, which leads to normalization of the biochemical indices (16). Several studies have suggested that this operation improved both QoL and muscle strength (17–19). However, with current changes in clinical presentation to mild to asymptomatic PHPT, surgery may not always be necessary (20).

HypoPT, instead, is a rare endocrine disorder caused by chronic deficiency or absence of PTH. The most common etiology of the disorder is the removal of, or injury to, the parathyroid glands during a complication of anterior neck surgery, for example total thyroidectomy or radical neck dissection for head and neck malignancies. Other etiologies include autoimmune, genetic and, very rarely, infiltrative disorders (such as hemochromatosis) (21, 22). Hypocalcemia, hyperphosphatemia, and low or undetectable PTH levels can cause muscle symptoms, such as cramps, seizures, paresthesias and numbness, life-threatening arrhythmias, laryngospasm, and bronchospasm. Chronic deficiency of PTH leads to abnormalities in bone microarchitecture, density, and strength, as a result of a profound reduction in bone remodeling. Many patients with HypoPT complain of reduced QoL, in particular, describing symptoms of cognitive dysfunction, described with the term “brain fog” (23).

Myopathy of the skeletal muscle, characterized by increased serum levels of creatine kinase (CK) and histological abnormalities in muscle biopsies, is also seen in HypoPT, and seems to relate to the severity of hypocalcemia (23, 24). Increased levels of serum CK are indicative of muscle injury and muscular dystrophy (25). Myopathy with elevated CK enzyme levels is a rare manifestation of HypoPT, first reported in 1972 (26); since then, there has only been a small number of reports describing this association (27).

Calcium supplement and active vitamin D are the conventional treatment of HypoPT and can control the serum calcium levels, but high doses may be required, exposing patients to the risk of long-term soft tissue calcifications.

Replacement therapy with recombinant human PTH (1–84) represents a major step in the therapies of this disease (28). In fact, PTH (1–84) is a more attractive replacement hormone in HypoPT because the full-length peptide is the missing hormone of this disease. Therapy with PTH peptides makes it possible to reduce calcium and active vitamin D administration with an improvement of neuromuscular symptoms vs. treatment with the standard therapy (29). The reasons for this effect are still unknown, but it has stimulated research into new treatment modalities and novel PTH analogs to uncover potential mechanisms of PTH on neuromuscular tissues.

Clinical trials demonstrating the efficacy of PTH for the treatment of HypoPT have led to the 2015 approval by the FDA in the United States of full-length recombinant human PTH [rhPTH (1–84)] for the management of hypoparathyroidism. Many clinical studies regarding its impact on QoL and effect on long-term complications are still ongoing (21, 30–32).

## PTH, Mineral Homeostasis and Its Receptors

The parathyroid glands control extracellular calcium homeostasis by secreting PTH. In the parathyroid cells, PTH is synthetized as a precursor peptide of 115 amino-acids (pre–pro-PTH), which later matures and is cleaved into full-length PTH, a single-chain polypeptide of 84 amino-acids, and stored in granules that can be secreted by the parathyroid glands when circulating ionized calcium concentrations are reduced. The biological activity of the intact PTH is associated to the 1–34 amino-terminal portion (33).

Changes in serum calcium levels are detected by the Calcium-Sensing Receptor (CaSR), a G-protein-coupled receptor that is highly expressed on parathyroid cell membrane. A decrease of extracellular ionized calcium concentration (hypocalcemia) induces a marked increase in PTH synthesis and secretion from the parathyroid glands. The secreted PTH circulates in the bloodstream and acts on bone and kidneys to increase serum calcium levels, which leads to feedback inhibition of PTH secretion from the parathyroid glands (23). Moreover, PTH stimulates the kidney tubules to produce the most active form of vitamin D, calcitriol (1,25-dihydroxyvitamin D) from calcidiol (25-hydroxyvitamin D), a less active form of vitamin D. Calcitriol stimulates the absorption of calcium from the gastrointestinal tract, helping increase serum calcium concentrations.

PTH also regulates circulating phosphate levels. In fact, it inhibits the tubular phosphate reabsorption by the kidney, thereby decreasing serum phosphate concentrations (34). Therefore, PTH is the major mediator of calcium and phosphate metabolism, and it carries out its action through interactions with receptors in two principal target organs, kidney and bone.

PTH receptor type 1 (PTHR1) is a G-protein-coupled receptor through which PTH (1–84) mediates its classical actions. This receptor is expressed in bone, on osteoblast and osteocyte surfaces, and in tubular cells in the kidney, but it is also present in other tissues, such as mammary glands and intestine (35). Stimulation of the PTHR1 leads to the Gα_s_-mediated activation of the adenylyl cyclase/cyclic AMP (cAMP)/protein kinase A (PKA) signaling pathway. The PTHR1 is also coupled to Gα_q_-mediated activation of the Gα_12/13_-phospholipase D/RhoA pathway, the phospho-lipase/protein kinase C (PKC) signaling cascade, and the extracellular signal-regulated kinase 1/2 (ERK1/2) / mitogen-activated protein kinase (MAPK) signaling cascade, the latter via G protein–dependent and G protein–independent/β-arrestin–dependent mechanisms (36).

PTHR1 is also activated by PTH-related peptide (PTHrP), first isolated in 1987 in the search for the causative agent of hypercalcemia of malignancy (37). The amino-terminal sequence of both PTHrP and PTH, constituted by the first 34 amino-acids, interacts with the same receptor, PTHR1, and is necessary for its action, stimulating the above pathways at target tissues.

Although both PTH and PTHrP signal via the same receptor, the biological functions of the two ligands are distinct, as PTH acts in an endocrine manner on bone and kidney cells to regulate blood levels of calcium and phosphate, enhancing bone remodeling, as evidenced by slow bone turnover in individuals with PTH deficiency (38), whereas PTHrP, in normal physiology, acts in a paracrine manner within developing tissues, such as the skeletal growth plate, to regulate osteoblast and chondrocyte differentiation and proliferation, but also in breast cells and skin (37, 39).

In humans, PTH potently activates a second PTH receptor, called PTHR2, leading to cAMP accumulation, whereas PTHrP is almost inactive. This receptor is abundantly expressed in the central nervous system (hypothalamus), but also in placenta and pancreas (40).

Moreover, a third PTH-recognizing receptor has been identified in zebrafish and broadly shares the same pharmacological profile with PTH1R (40).

## Skeletal Muscle as A Target Organ for PTH

Bone and skeletal muscle interact anatomically and biochemically with each other, and they are considered to be a single functional system whose relationship is essential for the physiology of the entire body. PTH is fundamental for the maintenance of calcium homeostasis through its actions to regulate bone remodeling, and the catabolic and anabolic actions that PTH exerts on the skeleton are well known and reported. This hormone can promote both bone formation and bone resorption by direct effects on osteoblasts and indirect actions on osteoclasts. The final effect on bone mass depends on the duration and periodicity of PTH exposure; bone resorption predominates when continuous exposure to high levels of PTH persists, whereas administration of low and intermittent doses of PTH leads to a net increase in bone mass (41, 42).

Since muscle is functionally linked to bone, it is likewise important to investigate the effects of PTH treatment on muscle tissue. It is indeed conceivable that variation in serum calcium concentration can affect skeletal muscle tissue, also considering the well-known involvement of calcium in muscle contraction (43).

It has been reported that both intact PTH and its amino-terminal fragment carry out their action by enhancing proteolysis and increasing the release of alanine and glutamine; these activities lead to an alteration of amino-acid metabolism and consequently affect muscle protein metabolism (44). Moreover, PTH administration causes biochemical derangements, effects of which are responsible not only for protein metabolism tampering but also bioenergetic alterations in the skeletal muscle. Reduced ATP content, activity of mitochondrial Mg ATPase, and mitochondrial oxygen consumption all participate in the consequent decrease of energy production in skeletal muscle (45).

Excess of PTH appears to have detrimental effects of skeletal muscle metabolism, since it has been shown that PHPT is associated with muscle dysfunctions, such as muscular weakness, myopathy and postural stability (46–48). Pattern et al. (49) described, in primary PHPT, atrophy of muscles, particularly in the lower extremities: muscle biopsies evidenced atrophy of both type I and type II muscle fibers, with type II fibers more extensively involved. Furthermore, it has been reported that in muscle biopsies from primary PHPT, where sustained stimulation of PTH is present, profound alterations in muscle gene expression are found, which may help explain symptoms and clinical PHPT manifestations, such as muscular fatigue (13).

Levels of PTH increase with age, and it has been hypothesized that the age-related change in hormone concentration may be responsible for the loss of muscle strength and muscle mass, characteristic in sarcopenia (50). Evidence has highlighted that PTH and PTHrP stimulate white adipose tissue browning through the activation of PKA, which was found to activate thermogenic and atrophy-related genes, among which the uncoupling protein-1 (UCP-1), atrogin-1, and muscle RING-finger protein-1 (MuRF-1). Moreover, an increase of PTH/PTHrP appears to upregulate the ubiquitin-proteasome proteolytic system (UPS) to degrade muscle protein and, consequently, leads to muscle wasting (51).

Although bone and kidney are the classical PTH targets, associated with calcium homeostasis, where mRNAs of PTH receptors have been found to be highly expressed, numerous reports demonstrate a wide tissue distribution of the PTH receptors in non-traditional targets for the hormone, such as heart, liver, brain, pancreas, and others. In these tissues, it has been demonstrated that PTH can affect cell function, since acute exposure of the hormone results in cAMP accumulation, suggesting that their response to PTH involves an interaction between the hormone and its receptors (52, 53).

Research on skeletal muscle in recent years has evidenced the importance of this tissue as a secretory organ, which, thanks to the production and release of myokines into the blood stem, can interact with other organs and tissues (7, 54, 55).

**Figure 2:**
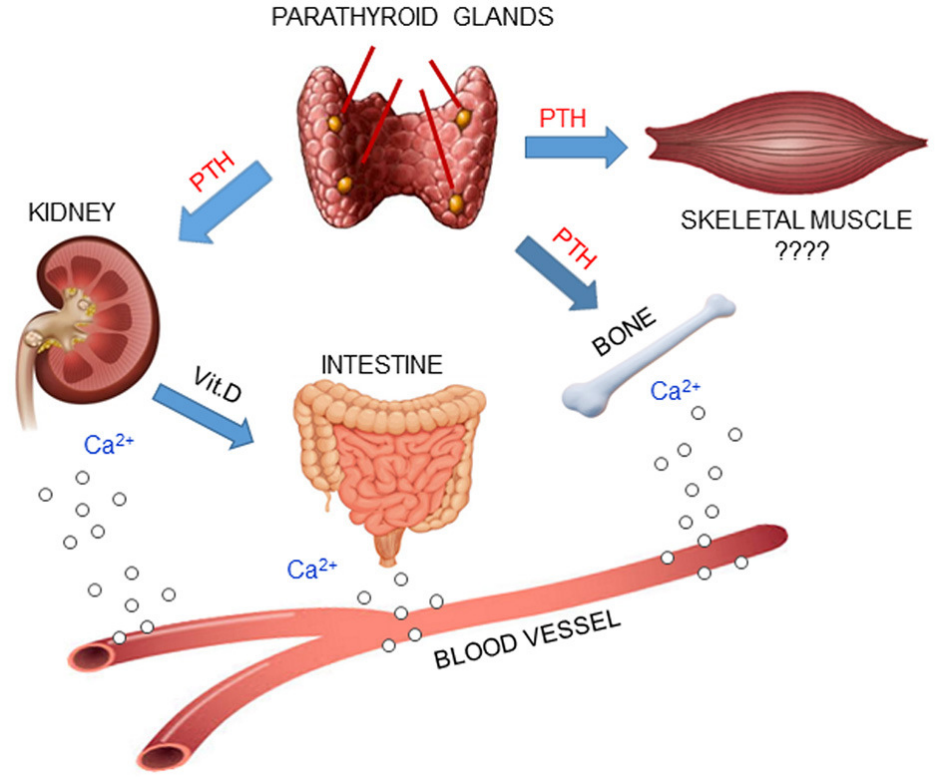
PTH plays a central role in mineral homeostasis and kidney and bone are the traditional targets of this hormone. When calcium levels in the blood decrease, parathyroid glands release PTH, which promotes calcium mobilization from bone, reabsorption in the kidney and absorption in the small intestine, *via* activation of Vit.D. Since PTH receptors have been found in skeletal muscle, it is possible that PTH may also have an effect in that tissue.

Skeletal muscle is a target organ for several hormones, such as insulin and estrogens. PTH may be one of these hormones, mediating its actions, which are distinct from the hormone's traditional function as regulator of mineral homeostasis (56, 57) (Figure 2). This hypothesis is supported by molecular evidence that mRNAs of PTH receptors, both PTHR1 and PTHR2, are expressed in skeletal muscle (13, 40, 53, 58).

The identification of PTHR1 and PTHR2 mRNAs in the brain may indicate that a lack/excess of PTH stimulation within in this organ can explain some of the neuropsychiatric manifestations, cognitive disorders, and reduced QoL in patients with HypoPT and PHPT (13).

Although abundant clinical evidence suggests an effect of PTH on skeletal muscle tissue, there has been little basic research done regarding *in vitro* studies on the effect of this hormone on the skeletal muscle. In particular, *in vitro* human cell models represent a valuable tool to contribute to the comprehension, at cellular and molecular levels, of the effects of PTH on skeletal muscle cells, in order to identify new mechanisms of action that may control skeletal muscle differentiation.

A recent article by Sato et al. reports the effects of the human recombinant PTH (1–34) on bone and skeletal muscle in a rat model of osteoporosis and muscle atrophy. Data showed that monotherapy with PTH (1–34), dosage of 30 μg/kg body weight three times per week, significantly increases the proximal and distal femoral bone mineral density (BMD) and the percentage of skeletal muscle mass in the ovariectomized, tail-suspended rats (59). Another article reported that PTH (1–34) and growth hormone prevent disuse osteopenia and sarcopenia in rats (60) and, in addition, PTH (1–34) treatment significantly improves muscle endurance in dystrophin-deficient mdx mouse (61). Although the effects of PTH (1–34) remain unknown, it may have an anabolic effect on muscle atrophy and muscle weakness. In any case, further investigation is needed.

Kimura et al. (62) reported, for the first time, the role of PTH in skeletal muscle regeneration, evidencing in a mouse cellular model, the importance that both PTHR1 expression and PTH (1–34) have to induce myocyte differentiation, accelerating myogenesis and myotube production.

Another *in vitro* study reports that the presence of PTH receptors was demonstrated by immunohistochemistry and by western blot in differentiated myotubes and on muscle fibers. Moreover, PTH (1–34) modulates the uptake and retention of 25-hydroxyvitamin D in the mouse C2C12 skeletal muscle cells differentiated in myotubes (63). Moreover, *in vitro* studies on C2C12 treated with PTH (1–34) significantly downregulated the expression of fibronectin type III domain-containing protein 5 (FNDC5), the precursor of irisin, suggesting an inverse interplay between the metabolism of these two important molecules (64).

In Table 1 are summarized the *in vivo/in vitro* studies on the role of PTH on skeletal muscle.

**Table 1:** Studies on the effects of PTH on skeletal muscle in *in vivo/in vitro* models.

**Species**	**Type of study**	**Treatment**	**Effects**	**References**
Rat	*In vivo*	PTH (1–34)	Increase in muscle vascularization	(25)
Rat	*In vitro*	PTH (1–84)	Enhance in skeletal muscle proteolysis	(44)
		PTH (1–34)		
Rat	*In vivo*	PTH (1–84)	Impair in skeletal muscle energy metabolism	(45)
		PTH (1–34)		
Mouse	*In vivo/In vitro*	PTH (1–84)	Increase in muscle wasting and muscle protein degradation	(51)
		PTH (1–34)		
Rat	*In vivo*	PTH (1–34)	Increase in skeletal muscle mass	(59)
Rat	*In vivo*	PTH (1–34)	Prevent sarcopenia	(60)
Mouse	*In vivo*	PTH (1–34)	Increase in muscle strength	(61)
Mouse	*In vitro*	PTH (1–34)	Induce in myocyte differentiation and myotube production	(62)
Mouse	*In vitro*	PTH (1–34)	Modulate Vit.D uptake	(63)
Mouse	*In vitro*	PTH (1–34)	Downregulate FNDC5	(64)

Our research group recently developed an *in vitro* model of skeletal muscle cells isolated from human biopsies of healthy donors who underwent reconstructive plastic surgery not attributable to endocrine diseases, to characterize skeletal muscle endocrine machinery and its hormonal regulation. The established myogenesis model used for the analysis has allowed us to highlight significant increases in PTHR1 gene expression during satellite cell differentiation, supporting the possible involvement of PTH in skeletal muscle regeneration and function, and confirming the utility of myogenesis models as a crucial factor for the development of possible new therapies (65).

## Future Insights

Considering the presence of PTH receptors in many non-traditional target organs for the hormone, even skeletal muscle, it is evident how important it is to increase efforts to understand how PTH acts, not only on the regulation of bone and kidney cells, but also on skeletal myogenesis. Numerous studies have shown that the proliferation and differentiation of satellite cells during muscle regeneration are profoundly influenced by innervation, vasculature, hormones, nutrition, and extent of tissue injury (66). Future studies, including *in vitro* analysis at the molecular and cellular levels, are therefore necessary for better and more specific comprehension of the effects of PTH on skeletal muscle cell proliferation and differentiation.

Furthermore, since the bone and skeletal muscle units are in a close relationship, the study of the actions of PTH on the muscle compartment could open new windows to understanding the interactions between these two important tissues and enable the development of a PTH therapy for the diseases characterized by skeletal muscle degeneration, such as HypoPT.

## Author Contributions

CR and MB contributed equally to the writing of the manuscript. Both authors have read and agreed to the published version of the manuscript.

## Conflict of Interest

The authors declare that the research was conducted in the absence of any commercial or financial relationships that could be construed as a potential conflict of interest.

## Publisher's Note

All claims expressed in this article are solely those of the authors and do not necessarily represent those of their affiliated organizations, or those of the publisher, the editors and the reviewers. Any product that may be evaluated in this article, or claim that may be made by its manufacturer, is not guaranteed or endorsed by the publisher.
